# A New Targeted Therapy Strategy for Lymphoma

**DOI:** 10.1002/mco2.70529

**Published:** 2025-11-28

**Authors:** Robert Peter Gale

**Affiliations:** ^1^ Centre for Haematology Imperial College of Science, Technology and Medicine London UK

1

In a recent article in *Signal Transduction and Targeted Therapy*, Zhao et al. from Ruijin Hospital, Shanghai reported results of the GUIDANCE‐06 trial testing a molecular subtype‐guided therapy strategy in relapsed/refractory diffuse large B‐cell lymphoma (DLBCL) [1].

They used a genetic sub‐typing algorithm to classify lymphomas in which different targeted drugs were added to the standard R‐ICE (rituximab, ifosphamide, carboplatin, etopside) regimen: (1) zanubrutinib for the MCD‐like and BN2‐like sub‐types; (2) lenalidomide for the N1‐like and NOS sub‐types; (3) chidamide for the EZB sub‐type; (4) decitabine for the *TP53‐*variant subtype; and (5) tofacitinib for the ST2 sub‐type (Figure 1A). The overall response rate was 76% (95% Confidence Interval [CI], 67, 86%), and the complete response rate, 57% (45, 68%). For comparison, overall and complete response rates in the CORAL trial were 64% (57, 71%) and 37% (95% CI not reported). The ORCHARRD and NCIC‐CTG LY.12 trials using R‐DHAP (rituximab, dexamethasone, cytarabine, cisplatin) reported complete response rates of 22% (16, 28%) and 14% (95% CI not reported). Two‐year progression‐free survival (PFS) and survival in the GUIDANCE‐06 trial were 69% (57, 79%) and 88% (78, 94%), both better compared with the CORAL and ORCHARRD trials. Two‐year survival in subjects achieving complete or partial remission was similar regardless of whether or not they received a haematopoietic cell transplant. These data indicate the efficacy of combining R‐ICE and selected targeted therapies followed by lenalidomide maintenance therapy in relapse or refractory DLBCL, especially in people unsuitable for an autotransplant.

**FIGURE 1:**
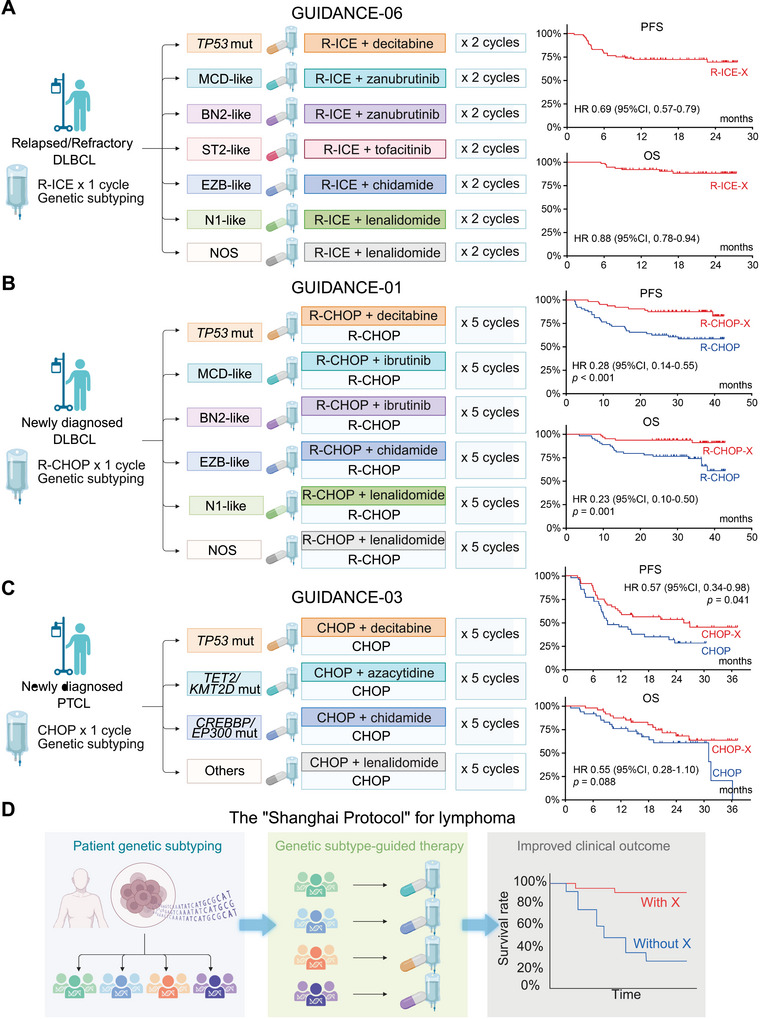
The “Shanghai Protocol” for lymphoma: a unified GUIDANCE paradigm systematically applied across the lymphoma spectrum. Schematic overview of the experimental designs for three key clinical trials in the GUIDANCE program, demonstrating a cohesive, biomarker‐driven therapeutic philosophy—termed the “Shanghai Protocol”—that is adapted across different clinical contexts and lymphoma subtypes, including R/R DLBCL (A, GUIDANCE‐06), newly diagnosed DLBCL (B, GUIDANCE‐01), and newly diagnosed PTCL (C, GUIDANCE‐03). Kaplan–Meier curves display progression‐free survival and overall survival for the R‐ICE‐X cohort and compare subtype‐guided therapy (R‐CHOP‐X, CHOP‐X) against standard chemotherapy in their respective trials, demonstrating the clinical benefit of this approach. (D) Conceptual framework illustrating the rational strategy: patient genetic subtyping leads to guided therapy and improved clinical outcomes. Survival curves in panels (A), (B), and (C) are redrawn based on data reported in the original publications [1, 2]. This figure is created with BioRender.com. *Abbreviations*: CHOP, cyclophosphamide, doxorubicin, vincristine, and prednisone; CI, confidence interval; DLBCL, diffuse large B‐cell lymphoma; HR, hazard ratio; OS, overall survival; PFS, progression‐free survival; PTCL, peripheral T‐cell lymphoma; R‐CHOP, rituximab with CHOP; R‐ICE, rituximab, ifosfamide, carboplatin, and etoposide.

The GUIDANCE‐06 trial was based on the results of two prior GUIDANCE studies. GUIDANCE‐01, a Phase‐2 randomized controlled trial (RCT) of initial therapy of intermediate‐ and high‐risk DLBCL [2]. GUIDENCE‐01 evaluated a genetic sub‐type‐guided strategy like the one used in GUIDENCE‐06. Targeted drugs were added to R‐CHOP (rituximab, cyclophosphamide, doxorubicin, vincristine, prednisone) including: (1) ibrutinib for the MCD‐like and BN2‐like sub‐types; (2) lenalidomide for the N1‐like and NOS sub‐types; (3) chidamide for the EZB sub‐type; and (4) decitabine for the *TP53*‐variant sub‐type (Figure 1B) [3]. Adding these drugs increased the complete response rate from 66% (54, 78%) to 88% (79, 96%; *p* = 0.003) and 2‐year PFS from 63% (49, 73%) to 88% (77, 94%; *p* < 0.001) and 2‐year survival from 77% (64, 85%) to 94% (84, 98%; *p* = 0.001).

The Phase‐2 GUIDANCE‐03 trial in peripheral T‐cell lymphoma (PTCL) added targeted drugs to the CHOP regimen including: (1) decitabine for the *TP53‐*variant sub‐type; (2) azacitidine for the *TET2*/*KMT2D*‐variant sub‐type; and (3) chidamide for the *CREBBP*/*EP300*‐variant sub‐type (Figure 1C). This strategy increased the complete response rate from 33% (20, 47%) to 65% (52, 78%; *p* = 0.004) and increased median PFS from 9 months (3.5, 14.5 months) to 26 months (8.5, 42.5 months; *p* = 0.04).

Every new innovation needs words of caution. The studies I discuss have design limitations and lack long‐term efficacy data. For example, GUIDANCE‐06 is a non‐randomized trial, a lower level of evidence compared with a randomized controlled trial (RCT). Median PFS in GUIDANCE‐03 and 2‐year PFS and survival in GUIDANCE‐06 need longer follow‐up to critically compare results of the “Shanghai Protocol” with alternative therapies. More data on adverse events are needed. Lastly, it is unclear how precision therapy promoted in the “Shanghai Protocol” would be combined with immune therapies.

Absent these caveats the GUIDENCE studies reflect the novel strategy of the “Shanghai Protocol”, a systematic, rational approach targeting variant drivers of lymphomas (Figure 1D). Data from the GUIDENCE trials suggest this strategy is effective in different lymphomas including DLBCL and PTCL, different disease states, initial and subsequent therapies and with different therapy backbones including CHOP, R‐CHOP and R‐ICE. The “platform trial” design is an innovative model for modern translational research and is being studied in prospective clinical trials globally [4, 5]. The strategy is dynamic, able to integrate new targeted drugs and immune therapies including antibody‐drug conjugates (ADCs), bi‐specific antibodies, chimeric antigen receptor (CAR)‐T–cells, and immune checkpointinhibitors. The “Shanghai Protocol” offers a new way to translate the promise of precision medicine into improved cancer outcomes.

## Author Contributions

RPG analyzed the literature, developed the typescript, approved the final iteration, accepts responsibility for the content and agreed to submit for publication.

## Funding

None.

## Ethics Statement

The author has nothing to report.

## Conflicts of Interest

RPG is a consultant to NexImmune Inc. Nanexa Pharma Ascentage Pharm Group and Antengene Biotech LLC, Medical Director of FFF Enterprises Inc.; Partner in AZAC Inc.; Board of Directors of Russian Foundation for Cancer Research Support and Scientific Advisory Board: StemRad Ltd.

## Data Availability

Not applicable.

## References

[1] Y. G. Shen , Q. Shi , W. Tang , et al., “Genetic Subtype‐Guided Immunochemotherapy in Relapsed and Refractory Diffuse Large B Cell Lymphoma: A Phase 2 Investigator‐Initiated Nonrandomized Clinical Trial (Guidance‐06),” Signal Transduction and Targeted Therapy 10, no. 1 (2025): 232. 40715072 10.1038/s41392-025-02316-6 PMC12297320

[2] M. C. Zhang , S. Tian , D. Fu , et al., “Genetic Subtype‐Guided Immunochemotherapy in Diffuse Large B Cell Lymphoma: The Randomized Guidance‐01 Trial,” Cancer Cell 41, no. 10 (2023): 1705–1716.e5. 37774697 10.1016/j.ccell.2023.09.004

[3] R. Shen , D. Fu , L. Dong , et al., “Simplified Algorithm for Genetic Subtyping in Diffuse Large B‐Cell Lymphoma,” Signal Transduction and Targeted Therapy 8, no. 1 (2023): 145. 37032379 10.1038/s41392-023-01358-y PMC10083170

[4] M. Mingalimov , E. Baryakh , D. Ivanova , et al., “Personalized Genetically Adapted Therapy for Newly Diagnosed Diffuse Large B‐Cell Lymphoma: A Single‐Center Prospective Study,” poster presented at European Hematology Association 2025 Congress (virtual), June 12, 2025.

[5] J. H. Liang , H. R. Shen , H. Yin , et al., “Novel Targeted Agents in Combination With R‐Mine (Rmine+X) Based on Different Molecular Subtypes in Relapsed/Refractory Diffuse Large B‐Cell Lymphoma: A Phase 2 Multicenter Study,” Blood 144, no. S1 (2024): 1725–1726.

